# Infantile Tumoral Calcinosis of the Cervical Spine: A Case Report

**DOI:** 10.5435/JAAOSGlobal-D-18-00062

**Published:** 2019-03-26

**Authors:** William M. Steward, Ryan C. Roubion, Joseph A. Gonzales

**Affiliations:** From the Louisiana State University Health Sciences Center—School of Medicine (Steward); the Department of Orthopaedics (Dr. Roubion), Louisiana State University Health Sciences Center; and the Department of Pediatric Orthopedics (Dr. Gonzales), Children's Hospital of New Orleans, New Orleans, LA.

## Abstract

Tumoral calcinosis (TC) is an exceedingly rare disease, significantly so when located in the spine. Here, we present a 4-month-old patient with decreased head control and range of motion of the neck for several weeks. CT and MRI demonstrated a calcified mass in the retropharyngeal area and surrounding C1/C2, and TC was suspected. The patient underwent surgical biopsy and aspiration, which confirmed TC. The purpose of this case report is to document a rare disease, significantly so when taking into account both the location of the lesion and the patient's age, and to detail the treatment and response.

Tumoral calcinosis (TC) is a rare condition characterized by calcium deposits in periarticular soft tissues. This term can be used to describe both primary occurrences and those that arise from a secondary disorder, most frequently from kidney disease. Primary TC is usually further separated into two classifications based on the metabolic phosphorous status, either hyperphosphatemic or normophosphatemic. TC usually manifests in the first two decades of life as a painless mass around a joint. The most commonly affected joints are the hip, elbow, and shoulder. Here, we present a case of a TC in the cervical spinal canal because of the rarity of its location and the lack of literature on these occurrences.

## Case Report

**Figure 1:**
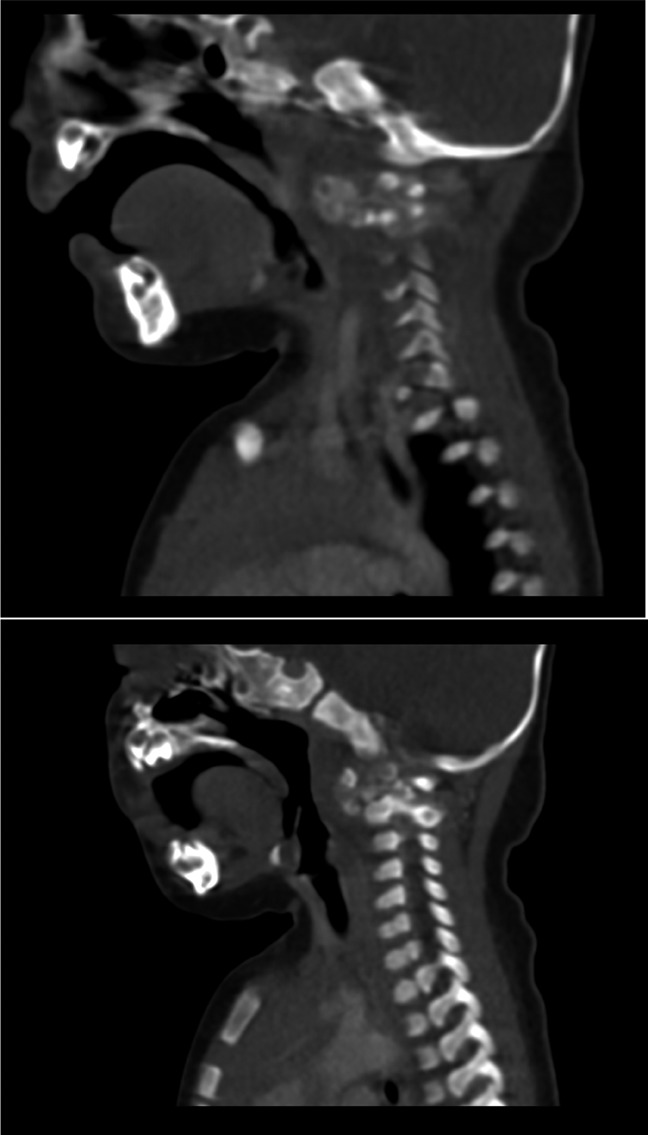
CT scan showing tumoral calcinosis. CT scan revealing the patient's tumoral calcinosis on initial presentation. Abnormal calcification and edema are seen in the retropharynx and around the C1/C2 vertebrae articulation.

A 4-month-old Caucasian female with a medical history of mild torticollis presented to the emergency department at Children's Hospital New Orleans on May 30, 2017, with loss of head control milestone and hypotonia of the neck for 2 weeks. She also had experienced decreased range of motion of the neck and poor feeding during this time. On examination, the patient was found to have poor head control and could not hold her head up when placed in either the upright position or the prone position. Range of motion and strength was normal in all extremities. Neck CT (Figure 1) and AP/lateral radiographs showed abnormal calcification and edema in the retropharynx and around the C1/C2 vertebrae articulation. MRI was also performed and supported these findings, showing enhancement and calcification in this area. TC was then suspected. Laboratory workup was then performed for secondary causes of TC, where parathyroid hormone, Ca, vitamin D, and P were all found to be within normal limits. Orthopaedic surgery and Ear, Nose, and Throat were consulted for operative management.

After imaging and discussion among the interdisciplinary team, it was agreed upon to take the patient for surgery using a biopsy procedure to confirm an accurate diagnosis of TC. After induction of anesthesia and orotracheal intubation, the patient was positioned in the operating room bed and mouth was opened with a Crowe-Davis mouth gag. A bulge was seen in the left retropharyngeal space, and an incision was made in the retropharyngeal space. A stat was then inserted and opened, and fluid with chalky white deposits was aspirated and sent for microbiology and pathology. A curet was then taken and the anterior portion of the lesion was curettaged, and about 2 mL of chalky white fluid mixed with serosanguinous fluid was aspirated, only taking the portions of the mass that were easily accessible. Afrin-soaked pledgets were packed into the incision to close the gap, and the patient was handed back to anesthesia for recovery.

**Figure 2:**
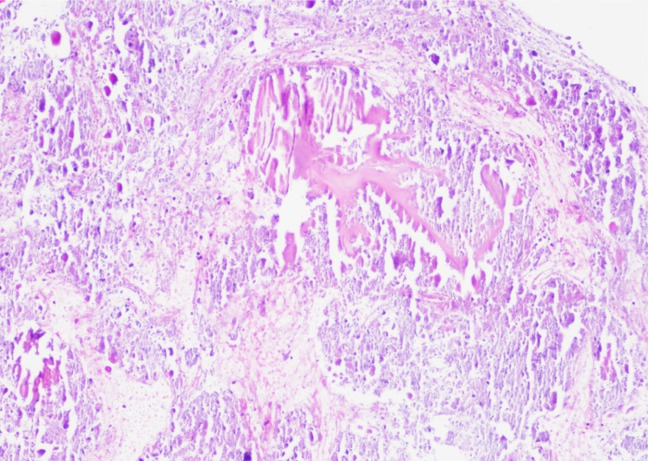
Histopathology slide of tumoral calcinosis. Low power view of biopsy aspirate showing calcified material, without bone lamellar architecture. The calcified material is blue and pink, acellular, without inflammation, and shows no granuloma. (Hematoxylin and Eosin, ×100 final magnification).

Pathology (Figure 2) demonstrated calcified material with scanty cellular material. Calcification was psammomatous in some areas with deposition of bluish and pinkish calcified material. No lamellar bone or increased inflammation was identified, nor was granulomatous material appreciated.

**Figure 3:**
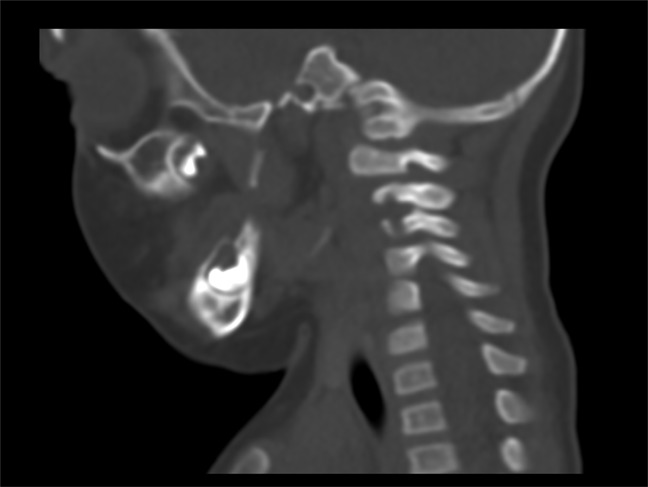
CT scan showing resolved tumoral calcinosis. CT scan of the patient at 11 months postoperatively showing resolution of the tumoral calcinosis.

The patient recovered well postoperatively and saw improvements with apraxia and head control during the hospital stay, as well recovered feeding capabilities, and was discharged on postoperative day 7. The patient was then followed up with serial CT scans and with neurology over the next 5 months. The patient returned to the clinic for follow-up at 5 months postoperatively and was found to have no neurological deficits. Full strength and range of motion was seen in the neck and all four extremities, and no sensory deficits were noted. Repeat CT showed no residual retropharyngeal calcifications, some persistent calcification surrounding dens and extradural space at C1 and C2 levels, and no new calcium deposits. A subsequent follow-up at 11 months postoperatively continued to show no physical complaints, and a normal examination and repeat CT (Figure 3) showed near complete resolution of TC.

## Discussion

TC is a rare disease with an unclear cause. As previously discussed, a classification system was developed by Smack et al^1^ after reviewing cases of TC; the pathogenesis-based classification system was proposed as follows: primary normophosphatemic TC, primary hyperphosphatemic TC, and secondary TC, most commonly from renal failure. Primary normophosphatemic TC is the most common form of the disease, and patients have no known disorders of calcium or phosphate metabolism, which was also demonstrated in this case of primary normophosphatemic TC. Trauma has been hypothesized to contribute to the development of the TC lesion because injury has been frequently reported preceding the development of TC, especially in the primary normophosphatemic form, and minimal repetitive trauma or chronic pressure has also been implicated.^2^ In this case, however, no preceding injuries or trauma were noted before the development of TC, which was further evidenced by the histopathologic evaluation of the biopsy specimen that revealed predominately calcified, acellular material with a notable absence of granulomatous tissue or bone marrow elements such as leukocytes. These inflammatory changes would normally be seen on pathology after muscle trauma^3^ or traumatic brain injury^4^ if they had occurred, thus the absence of such material provides further support to the atraumatic development of TC in this case.

TC most commonly involves the periarticular soft tissues of the large joints of the extremities, including the hip, elbow, and shoulder, as well as the foot and wrist less commonly.^5^ However, although not widely prevalent, TC involving the spine has been reported^6^ in the literature, along with cases specifically involving the cervical spine^7,8,9^ which was seen in this case. Regarding TC of the spine, a literature search revealed 15 cases of TC involving the cervical spine, not including this case, in addition to three involving the thoracic spine and 14 involving the lumbar spine.^9^ Of note, the youngest reported case of TC involving the spine was found to be 1.4 years of age,^10^ making this case possibly the youngest instance of a spinal primary TC reported in literature at 4 months of age.

TC most commonly manifests as a painless and asymptotic mass around joints, but it may cause compression symptoms, including pain, weakness, and sensory deficits, or limited range of motion of the joint involved.^11^ Here, the patient presented with loss of the head control milestone and decreased tone and range of motion of the neck.

Treatment for TC most frequently involves surgical resection, especially for symptomatic cases causing neurologic dysfunction or joint function limitations. With the primary forms of TC, surgical resection is the primary treatment and has been shown to be successful with low recurrence rates.^1,11^ Furthermore, surgical resection has been shown to be curative in cases of primary TC seen specifically in infants, with some cases additionally reporting evidence of spontaneous regression of lesions in this age group.^12^ A curative result was also seen in this case although resection was performed only of the accessible area for biopsy, and it successfully resulted in a return to normal functioning of head control and neck range of motion. The intraspinal area was then monitored and treated conservatively with serial imaging and a clinic follow-up. As of this writing, no occurrence of neurological or musculoskeletal deficits has been observed. In addition, serial imaging has demonstrated a near complete resolution of the TC with only residual punctate calcifications surrounding the dens, suggesting spontaneous regression of the nonresected portion of the lesion, and no incidence of new calcifications or deposits have been seen.

## Conclusion

TC is a rare disease, especially when located within the cervical spine, with an unclear etiology. However, it should be considered on the differential diagnosis when considering spinal lesions. Common presenting symptoms can include pain, weakness, sensory deficits, and limited range of motion in the involved joint. Surgical resection is the primary method of treatment. However, in this case, observation was the primary method of treatment after surgical biopsy, which should be considered in the rare instance of intraspinal lesions in the neonate or infant with a nondeteriorating examination.
